# Assignment of polymorphic species of insulin analogues in ion mobility mass spectroscopy

**DOI:** 10.1016/j.dib.2016.12.020

**Published:** 2016-12-21

**Authors:** Maely P. Fávero-Retto, Luiz Henrique Guerreiro, Cássio M. Pessanha, Leonardo C. Palmieri, Luís Maurício T.R. Lima

**Affiliations:** a Federal University of Rio de Janeiro – UFRJ, Av. Carlos Chagas Filho 373, CCS, Bss24, Ilha do Fundão, 21941-590 Rio de Janeiro, RJ, Brazil; b Laboratory for Macromolecules (LAMAC-DIMAV), Brazilian National Institute of Metrology, Quality and Technology - INMETRO, Av. N. Sa. das Graças, 50 - Xerém, Duque de Caxias 25250-020, Rio de Janeiro, Brazil; c National Institute of Science and Technology for Structural Biology and Bioimaging (INBEB-INCT), Federal University of Rio de Janeiro, Rio de Janeiro 21941-590, Brazil; d Brazilian National Cancer Institute (INCA), 20230-014 Rio de Janeiro, RJ, Brazil; e Department of Chemistry, Institute of Exact Sciences, Rural Federal University of Rio de Janeiro - UFRRJ, Rodovia BR 465, km 7, CEP:23890-000 Seropédica, RJ, Brazil

## Abstract

Electrospray ionization – ion mobility spectrometry – mass spectrometry (ESI–IMS–MS) allows the identification of protein polymorphic distribution of protein conformers and oligomers. We report the detailed identification of the species observed with commercially available pharmaceutical preparation of wild-type, regular human insulin.

**Specifications Table**

**Table:** 

Subject area	*Physics, Chemistry, Biology*
More specific subject area	*Structural Biology*
Type of data	*Figure (mass spectrometry)*
How data was acquired	*Mass spectrometry coupled to ion mobility spectrometry (Synapt HDMS G1, Waters Corp)*
Data format	*Analyzed*
Experimental factors	*Concentrated sample (human insulin 100 U/mL, 3.5 mg/mL, 600 μM) was diluted to 50 μM in 100 mM ammonium acetate.*
Experimental features	*Electrospray ionization - mass spectrometry coupled to ion mobility spectrometry measurements of regular-acting human insulin measured in ammonium acetate.*
Data source location	*Not applicable*
Data accessibility	*Data is with this article.*

**Value of the data**

• Proteins can populate a broad range of conformation and oligomeric states according to chemical and physical variables.
• Polymorphic distribution is a identity of a formulated biopharmarmaceutical product.
• Electrospray ionization mass-spectrometry coupled to ion-mobility spectroscopy (ESI–IMS–MS) allows separation of species by cross-sectional area.
• ESI–IMS–MS allowed the assignment of the diversity of oligomeric and conformational distribution of human insulin.
• ESI–IMS–MS may serve for the characterization of the effect of formulation on biopharmaceuticals products.

## 1 Data

Unidimensional mass spectrometry analysis of proteins, including biopharmaceuticals, does not allow a clear discrimination of polymorphic species with overlapping *m/z*. Oligomers sometimes can be observed the isotopic distribution, but can be limited in cases of low prevalence or multiple oligomeric states. Moreover, similar oligomeric state with same charged state sometimes may display multiple conformation, which can not be elucidated by unidimensional electrospray ionization mass spectrometry (ESI–MS). In such case, the separation of the species, either conformational or oligomeric, by ESI–MS coupled ion mobility spectrometry (ESI–IMS–MS) allow a more in-deep depiction of the complex polymorphic distribution in protein specimens, such as in biological, biopharmaceutical products

**Fig. 1:**
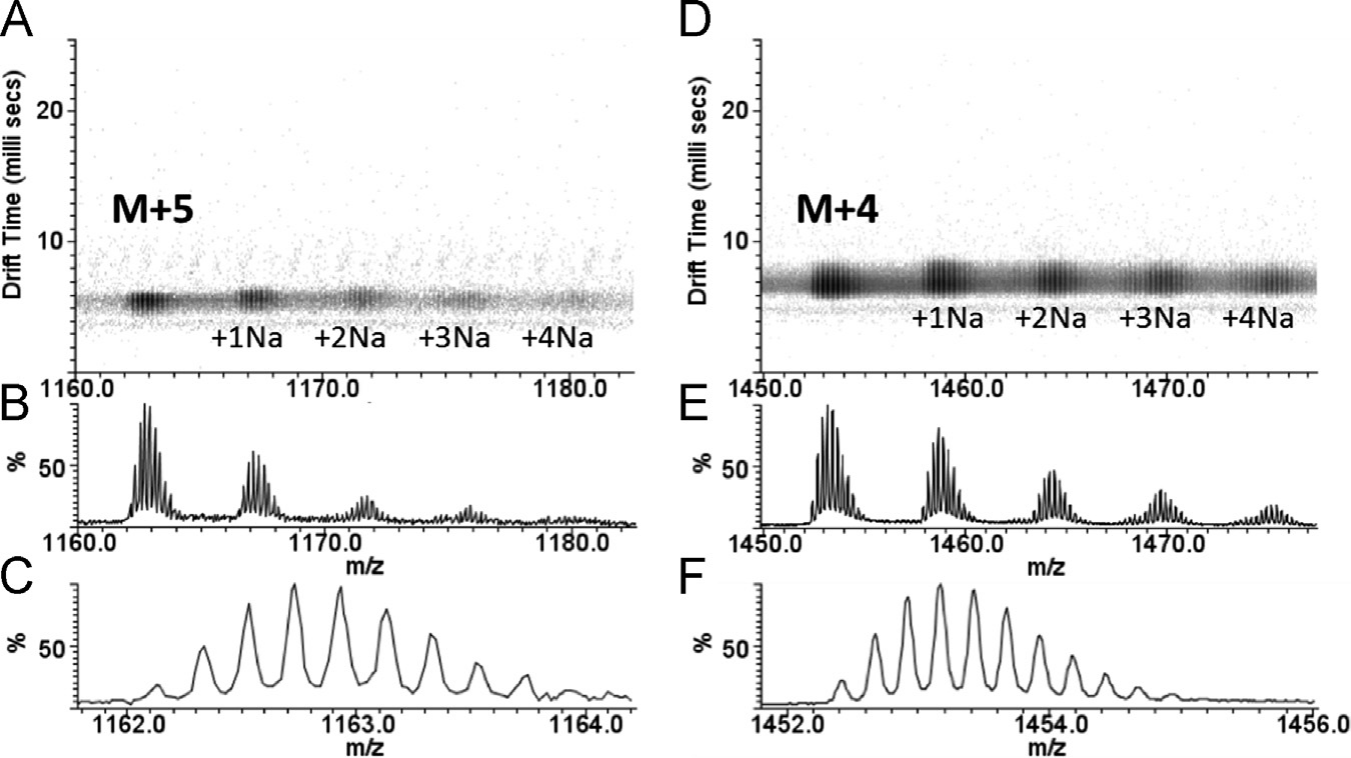
ESI–IMS–MS spectra of regular insulin – monomers. The monomer in the +5 charged state (A, B and C) and in the +4 charged state (D, E and F) are shown for their sodium adduct series (A, B, D and E), and no evidence for two distinct conformational states are found based on the separation in the drift time axis (A and D). The isotopic distribution of the free monomer is shown for M+5 (C) and M+4 (F).

**Fig. 2:**
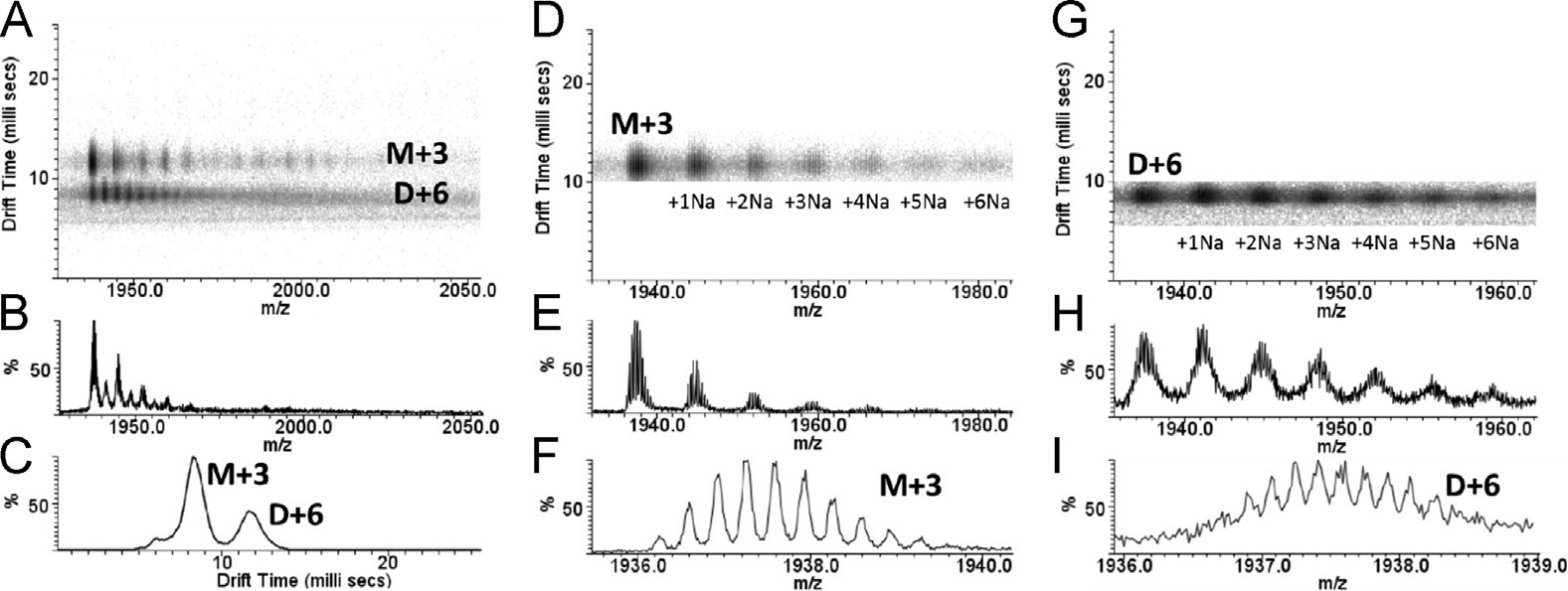
ESI–IMS–MS spectra of regular insulin – monomer and dimer. The M+3 and D+6 of insulin show coincident *m/z* signal, but are well separated in the drift time axis (A and C). Profiling stripping allowed the separation of the signal of the M+3 (D, E and F) and D+6 (G, H and I).

**Fig. 3:**
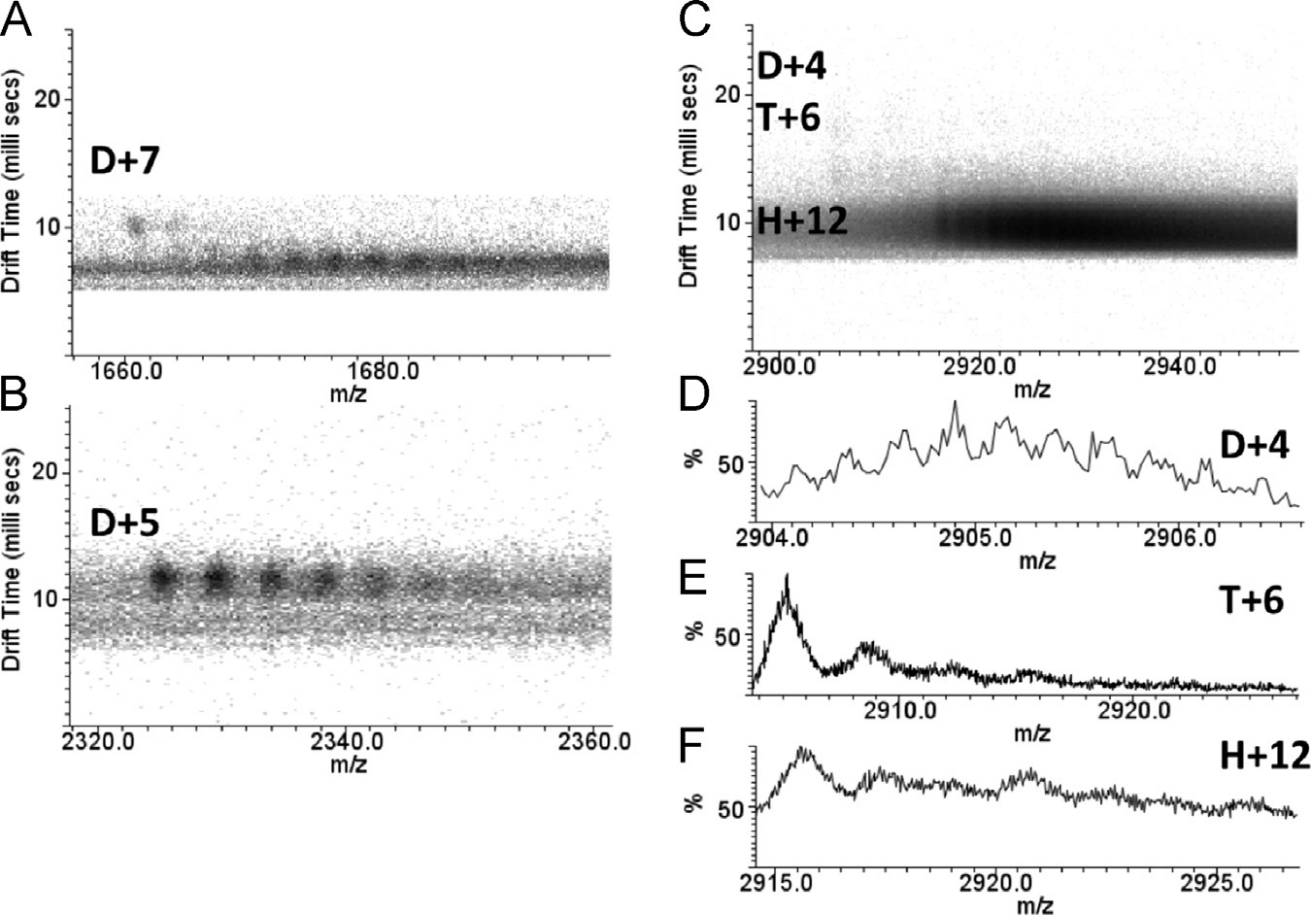
ESI–IMS–MS spectra of regular insulin – dimer, trimer and hexamer. The dt.*m/z* spectra of D+7 (A), D+5 (B), and coincident D+4, T+6 and H+12 (C) and their series of sodium adducts. The coincident D+4, T+6 and H+12 ions in the *m/z* axis can be separated in the dt axis (C) allowing the stripping in their respective spectra, respectively (D), (E) and (F).

**Fig. 4:**
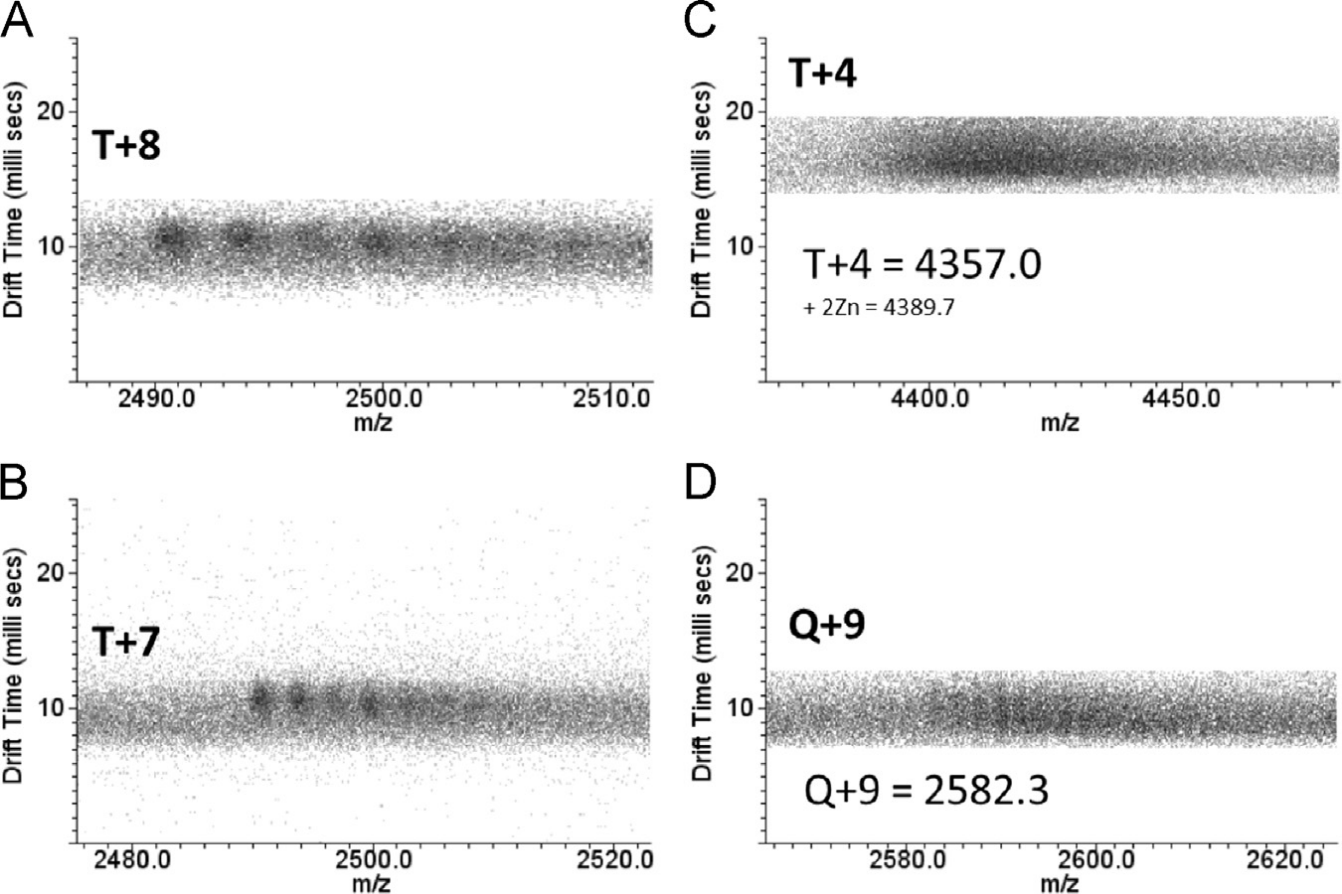
ESI–IMS–MS spectra of regular insulin – trimer and tetramer. The dt.*m/z* spectra of the (A) T+8, (B) T+7, (C) T+4 and (D) Q+9 charged states and their respective sodium adducts.

**Fig. 5:**
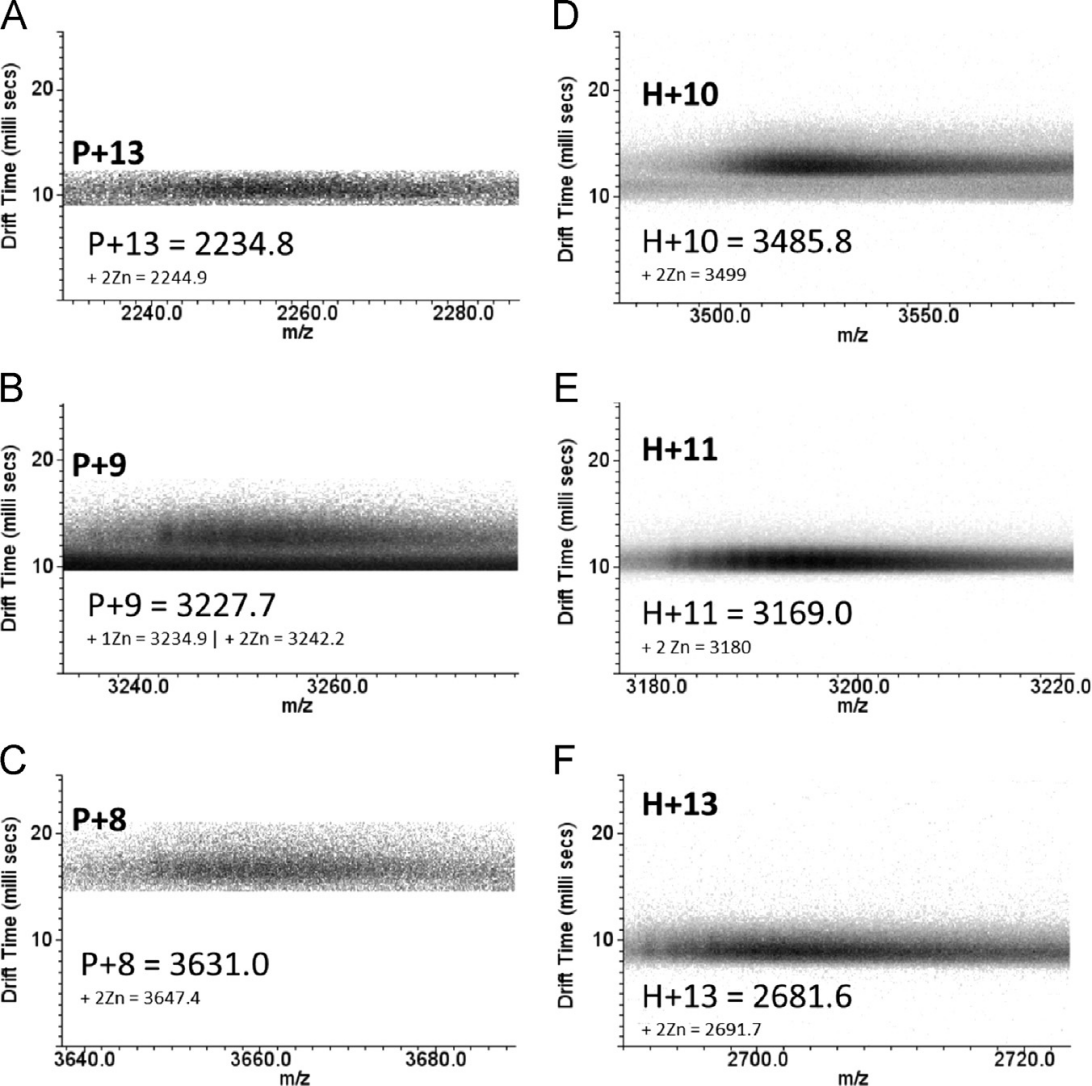
ESI–IMS–MS spectra of regular insulin – pentamer and hexamer. The dt.*m/z* spectra of zinc complexes of (A) P+13, (B) P+9, (C) P+8, (D) H+10, (E) H+11 and (F) H+13, and their respective sodium adducts.

**Fig. 6:**
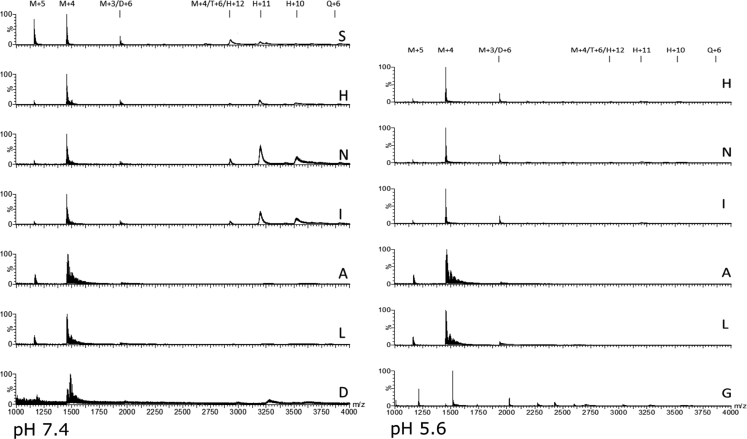
ESI–MS spectra of insulin at pH 7.4 and pH 5.6. S (Sigma-Aldrich, no formulation components), (H) regular Humulin R, (N) regular Novolin N, (I) regular (Insunorm R), (A) aspart (Novorapid), (L) lispro (Humalog), (D) detemir (Levemir), (G) glargine (Lantus). The list and relative intensity of the identified ions are depicted in Supporting Table S1.

Here we present data concerning the ESI–IMS–MS stripping of the drift-time spectra of commercial regular insulin, and from them we could identify monomers (Fig. 1, Fig. 2), dimers (Fig. 2, Fig. 3), trimers (Fig. 3, Fig. 4), tetramers (Fig. 4), pentamers (Fig. 5) and hexamers (Fig. 3, Fig. 5) and their respective sodium adducts (Fig. 6).

## 2 Experimental design, materials and methods

### 2.1 Chemicals

The pharmaceutical product regular human insulin (100 U/mL = 600 μM; Humulin R^®^; lot #C020193, #A560347A), was purchased from local pharmacy and stored at 4 °C – 8 °C until use. All other reagents were of analytical grade.

### 2.2 Electrospray ionization–Ion mobility spectrometry–Mass spectrometry (ESI–IMS–MS) measurements

ESI–IMS–MS measurements were conducted in a Synapt G1 (Waters, USA) high definition mass spectrometer (HDMS) quadrupole-travelling wave mass spectrometer. The insulin samples were prepared from the formulated stock solution (600 μM = 100 U/mL) by diluting 5 times (120 μM = 20 U/mL) directly with the 100 mM ammonium acetate buffers pH 7.4. Measurements were performed immediately after sample preparation. The insulin samples were measured in a positive ESI mode, with a capillary voltage of 2.8 kV and N_2_g at 0.4 bar. The samples were injected at a rate of 10 μL/min. Data were accumulated for 20 min with a 3 s acquisition time per point, over the range of *m/z* 500–4000. Other typical experimental settings involved sampling and extraction cone set respectively at 30 V/5.0 V, source temperature of 70 °C, and nanoflow gas pressure of 400 mbar. The trap (before IM cell) and transfer (after IM cell) cells voltage were set to 6 and 4 V, respectively. The cell pressures were controlled by argon gas, while IM separations were performed by using N_2_g, and transfer wave velocity of 248 m/s and transfer wave amplitude set at 3.0 V. The mass calibration was performed on a dynamic mode by using H_3_PO_4_. All data were analyzed by using MassLynx 4.1 (Waters Corporation, Brazil) and DriftScope 2.4 (Williams, Lough 2009) (Waters Corporation, Brazil).
