# Supplementary material for: Assignment of polymorphic species of insulin analogues in ion mobility mass spectroscopy
Source: Data Brief. 2016 Dec 21;10:531–6. doi: 10.1016/j.dib.2016.12.020 (PMC5219646; doi:10.1016/j.dib.2016.12.020)
Supplement: Supplementary file 1 — Supplementary material [file mmc1.docx]

*Data article*

**Title:** *Assignment of polymorphic species of insulin analogues in ion mobility mass spectroscopy*

**Authors:** Maely P. Fávero-Retto^a,d^, Luiz Henrique Guerreiro^a,e^, Cássio M. Pessanha^a^, Leonardo C. Palmieri^a^ and Luís Maurício T. R. Lima^a,b,c*^,

**Conflict of Interest:** Prof. Luis Mauricio T. R. Lima is applicant of patents regarding controlled release of amylin.
