# Supplementary material for: Assignment of polymorphic species of insulin analogues in ion mobility mass spectroscopy
Source: Data Brief. 2016 Dec 21;10:531–6. doi: 10.1016/j.dib.2016.12.020 (PMC5219646; doi:10.1016/j.dib.2016.12.020)
Supplement: Supplementary file 2 — Supplementary material [file mmc2.pdf]

Table S1. Identification of insulin oligomers and ions by ESI-MS. (S) regular (Sigma-Aldrich, no formulation components), (H) regular Humulin R, (N) regular Novolin N, (I) regular (Insunorm R), (A) aspart (Novorapid), (L) lispro (Humalog), (D) detemir (Levemir), (G) glargine (Lantus). Numbers in Table correspond to the percentage of peak intensity of the ion of higher intensity in the charged state series in relation to the main ion (M+4).

pH 7.4

Rel Sig, %	M+5	M+4	M+3/D+6	D+7	D+5/Q+10	D+4/T+6/H+12	T+8	T+7	Q+9	Q+6	H+11	H+10
S	86.7	100.0	28.3	-	-	16.6	-	-	-	2.8	8.9	2.6
H	12.9	100.0	15.4	-	-	4.2	-	-	-	1.7	15.1	5.7
N	14.5	100.0	10.9	-	-	19.6	-	-	-	7.2	64.3	26.8
I	12.2	100.0	12.6	-	-	11.6	-	-	-	5.4	43.8	21.1
A	33.1	100.0	6.3	-	-	1.5	-	-	-	-	3.5	-
L	29.0	100.0	7.0	-	-	1.3	-	-	-	-	2.5	-
D	32.5	100.0	14.4	-	-	7.9	-	-	-	-	16.5	-

pH 5.6

Rel Sig, %	M+5	M+4	M+3/D+6	D+7	D+5/Q10	D+4/T+6/H+12	T+8	T+7	Q+9	Q+6	H+11	H+10
H	9.9	100.0	24.6	1.1	2.5	1.1	3.1	1.5	1.3	-	2.2	1.1
N	9.5	100.0	22.2	1.1	2.3	1.3	2.9	1.4	1.1	-	2.9	1.3
I	9.1	100.0	21.9	1.1	2.1	1.4	2.6	1.5	1.2	-	2.8	1.3
A	26.5	100.0	7.3	5.5	-	-	-	-	0.0	-	-	-
L	23.5	100.0	10.3	2.4	-	-	-	-	0.0	-	-	-
G	48.8	100.0	22.5	5.6	10.7	3.6	8.8	5.2	5.2	-	2.7	1.6
